# Demonstration of sizing nursing staff methods in intensive care

**DOI:** 10.1590/1518-8345.7184.4410

**Published:** 2025-01-27

**Authors:** Érica Batassini, João Lucas Campos de Oliveira, Mariur Gomes Beghetto

**Affiliations:** 1 Hospital de Clínicas de Porto Alegre, Serviço de Enfermagem em Terapia Intensiva, Porto Alegre, RS, Brazil.; 2 Universidade Federal do Rio Grande do Sul, Escola de Enfermagem, Porto Alegre, RS, Brazil.

**Keywords:** Personnel Downsizing, Workload, NursingTeam, Intensive Care Units, Nursing Staff Hospital, Critical Care

## Abstract

**Objective:**

to demonstrate the sizing of intensive care nursing staff estimated by two calculations, using the Nursing Activities Score as one of its central components.

**Method:**

descriptive, retrospective study that compiled the Nursing Activities Score scores of patients in five Intensive Care Units of a hospital in southern Brazil. Two calculations were used to size the nursing staff. In addition to other components, calculation I converted the Nursing Activities Score into minutes and hours; and calculation II used this score on a denominator corresponding to one nursing professional/day, considering two types of work shifts. In both equations, the proportion of 52% of nurses was respected.

**Results:**

in the 9,610 evaluations, the mean of Nursing Activities Score was 85.9% (80.8% to 96.4%). While calculation I estimated 164 professionals, calculation II projected 176 and 140 workers for five and four shifts, respectively. The difference in nurses between the methods was 18 professionals.

**Conclusion:**

the choice of the calculation and the number of work shifts substantially interfere in the estimation of intensive care nursing staff. This may support a methodological review in the standardization of nursing sizing in this area.

## Highlights

**(1)** The projection of intensive care nursing staff depends on the calculation method.

**(2)** The technical regulations on nursing staff in intensive care must be reviewed.

**(3)** The workload measurement instrument does not guarantee equal staff projection.

## Introduction

The evidence on the directly proportional relationship of increased nursing workload with worse clinical outcomes and patient safety outcomes is exponential, including increased chance of mortality ^(1-2)^. The elevation in workload also has an impact on the psychodynamics of nursing professionals, negatively affecting their job satisfaction and perception of exhaustion ^(3)^. In intensive care, this reality is confirmed and may even worsen ^(4-5)^, considering the greater clinical severity and the greater risk of healthcare-related infections, pressure injuries and medication errors due to the excessive workload, as confirmed by a systematic review ^(6)^.

An Iranian study ^(7)^ inferred that both the characteristics of patients (type and length of hospitalization, gender, and clinical or surgical admission) admitted to Intensive Care Units (ICU) and those of the nursing staff (shift, type of ICU, gender, and number of patients under care) affect the workload of these professionals. It is reiterated that workload is a complex construct to measure, and the “mere” number of patients per professional may not be the most reliable measure for its measurement, including in the ICU ^(4-5)^. The pandemic caused by the coronavirus was an example of this ^(8)^. However, the ratio of patients to nursing staff tends to negatively affect workers’ perception of their workload, also in intensive care ^(9)^.

In Portugal, a survey found that, on more than a third of the days analyzed (n=91), the work demand required of nursing was incompatible with the available human resources ^(10)^. This mismatch is also seen in Brazil, as the discrepancy between the different laws and guidelines regarding the practice of nursing in the care of critically ill patients constitutes a major obstacle to sizing the nursing staff for ICUs. In 2012, a Resolution from the Brazilian Federal Government established that ICUs must maintain a minimum ratio of one nurse for every 10 patients and one nursing technician for every two patients ^(11)^. On the other hand, for many decades, the Brazilian nursing professional practice law has determined that direct nursing care for seriously ill patients, with risk to life, is exclusive to nurses ^(12)^. Even so, the census conducted by the *Associação de Medicina Intensiva Brasileira* (AMIB) ^(13)^ identified that the number of nursing technicians working in ICUs is almost double the number of nurses.

The Nursing Activities Score (NAS) is internationally recognized as a sensitive and valid instrument for measuring nursing workload in ICU ^(4-10,14)^. The NAS is considered a better measure of workload than the Therapeutic Intervention Scoring System-28 (TISS-28) and the Nine Equivalents of Nursing Manpower Use Score (NEMS) ^(15)^. Despite this, the systematic and validated measurement of workload does not guarantee, by itself, the projection/sizing of nursing staff compatible with the reality of labor demand, since the sizing of nursing staff, although dependent, is a subsequent step of the workload measurement ^(16-17)^.

A scoping review ^(18)^ led by renowned English researchers in the area of nursing staff planning recommends that, despite the large volume of publications, evidence on nurse projection methods remains highly limited. The authors further note that there is no evidence to support the choice of any particular instrument and suggest that future research focus on learning more about the use of existing ones, rather than simply developing new ones ^(18)^.

In Brazil, the *Conselho Federal de Enfermagem* (COFEN) regulations on the parameters of staff planning and sizing, after legal demands, went from a resolution ^(17)^ to a technical opinion ^(19)^, which, despite including a sizing method for uninterrupted care units, such as ICUs, does not methodologically address the specificity of these peculiar care spaces. Variations in the measurement of intensive care nursing workload and, consequently, in staff projections are an obstacle to establishing the relationship between this variable and clinical outcomes ^(20)^, which are known to be valuable for improving critical care and also strengthening justifications for the adequacy of human resources.

There is difficulty in assertively predicting how many and which nursing professionals are needed to care for critically ill patients ^(5)^, which is in line with the idea that methods and strategies for estimating nursing human resources must be continually reviewed ^(16-18)^. Admitting that the estimation of nursing human resources in ICUs can compromise the quality of care, but also burden organizational sustainability, the objective of this study was to demonstrate the sizing of nursing staff in intensive care estimated by two calculations, using the NAS as one of its central components.

## Method

### Study design

Descriptive, quantitative and retrospective study. It was characterized by the analysis of primary data obtained through the computerized system of the research institution.

### Location

The study was carried out in a general Intensive Care Center (ICC) (except trauma) of a public university hospital with extra capacity in southern Brazil. The ICC has 45 beds distributed in five ICUs that receive adults with different characteristics, namely: ICU “A”: clinical patients with cardiovascular disorders and/or post-operative cardiac surgeries, with 10 beds; ICU “B”: surgical patients, with 10 beds; and ICUs “C”, “D” and “E”, which serve clinical patients of different specialties, with units C and D having 10 beds each, and E having 5 beds. There were no changes in the number of beds during the study period.

The ICUs nursing staff mostly works a 36-hour work week. The staff is distributed as follows: ICUs A, B, C and D have 10 nurses and 39 nursing technicians each, distributed across five work shifts. In ICU E, there are five nurses and 22 nursing technicians for the five shifts. In the general ICC staff, there are two support nurses for hemodialysis and 14 temporary nurses. There are also five support nursing technicians (one for each shift). There are no temporary nursing technicians.

### Period

The research covered the period from January to September 2023, enough time to size hospital units ^(17,19)^. Additionally, the period was defined after the declaration of the end of the public emergency related to COVID-19, since the study scenario was a reference for treating patients with this condition.

### Participants

All NAS values (n=9,610) computed during the study period were included, without exclusion criteria, obtained retrospectively through the institution’s computerized system.

### Study variables

The main variable of the study was the mean NAS score. The NAS is administered by ICC nurses during their care routines, as recommended by the original instrument translated and validated for Brazil ^(21)^. The NAS ^(21)^ is applied once a day, as well as at the time of the patient’s release (discharge or death), considering the events that occurred in the last 24 hours. Patients who die or are discharged from the ICC before 24 hours of hospitalization do not have their NAS scored. The 23 items, subdivided into seven domains (basic activities, ventilatory support, cardiovascular support, renal support, neurological support, metabolic support and specific interventions) of the NAS were evaluated. For each item, a specific score was assigned, with the maximum possible sum of all items being 176.8% ^(21-22)^. After the sum, the workload is classified, so that the percentage equal to 100% of NAS is equivalent to the demand for a nursing professional’s time 24 hours a day, for the care of a patient; 50 points is equivalent to 50% of a professional’s time and so on ^(22)^.

### Instruments used to collect data and information

After administering the NAS, nurses recorded the results in the hospital’s electronic patient management system. For the present study, data were obtained in an aggregated manner, stratified by ICU (A, B, C, D and E) and by month (January to September 2023). The corporate data analysis and metrics system (Business Analytics Strategic Intelligence - BASE^®^) was used to generate dynamic tables with the data, exported to a spreadsheet (Excel^®^). No individual data or data that would allow the identification of patients or nursing workers were collected.

### Data processing and analysis

Using the NAS score data, two staff sizing calculations were performed, now called Calculation I and II, the details of which are described in Figure 1. Furthermore, based on Calculation II, two alternatives for projecting professionals were produced: a) considering five teams/work shifts (morning, afternoon and three night shifts) and b) considering four teams/work shifts (morning, afternoon and two night shifts). This methodological definition was given because the field of study meets the first possibility (five work teams); however, it is widely known that the massive reality of Brazilian hospital nursing works with four work shifts. In both calculations, the Technical Safety Index (TSI) considered was a minimum of 15%, as recommended ^(16,19)^.

**Figure 1:** - Elements of Calculations I and II for the sizing of nursing staff in ICU*, using NAS^†^ as one of the components. Porto Alegre, RS, Brazil, 2023

**Elements**	**Calculation I**	**Calculation II**
Basis for measuring nursing workload and justification for sizing the nursing staff in the ICU*	NAS^†(21)^ mean of the unit in the period	NAS^†(21)^ mean of the unit in the period
Equation for sizing the ICU* nursing staff	**S‡ = TNH§ x MK|| (UAU¶)** Where: S^‡^: Staff; TNH^§^: Total Nursing Hours, obtained from the following equation: Total NAS^†^ points for the day x 14.4 minutes/60 MK^||^: Marinho’s Constant, coefficient deducted from the hours (WW**) and weekly days (WD^††^) worked (seven) and the Technical Safety Index (TSI^‡‡^)^(17)^. In this study, MK^||^ = 0.2236, compatible with a 36-hour working week and a minimum TSI of 15%. MK^||^_(UAU_^¶^_)_ = WD^††^ x (1 + TSI^‡‡^)^(17)^.	**NS§§= T|||| x (NAS† mean/100) + 15%** Where: NS^§§^: Nursing staff; T^||||^: number of ICU* teams (five or four). TSI^‡‡^: Technical Safety Index. In this calculation, TSI^‡‡^ = 15%.
Theoretical-scientific basis of the equation	Equation proposed by COFEN^¶¶(17)^ for uninterruptible assistance units (UAU^¶^), valid at the time of the study. Nursing hours extracted from converting the NAS^†^ score to minutes, in which each point equals 14.4 minutes^(22)^.	Proposal of the equation presented in a 2010 study^(23)^ and subsequent research that used it^(24-25)^. The value of 100 as the denominator of the NAS^†^ mean in the calculation equation refers to the basis that this is equivalent to the demand for a nursing professional’s time 24 hours a day^(22)^.
Percentage of nurses out of total sized staff	52%, assuming that the population treated in the ICU* is mostly intensive care patients^(17,19)^.	52%, assuming that the population treated in the ICU* is mostly for intensive care^(17,19)^.

*ICU = Intensive Care Unit; ^†^NAS = Nursing Activities Score; ^‡^S = Staff; ^§^TNH = Total Nursing Hours; ^||^MK = Marinho’s Constant; ^¶^UAU = Uninterruptible Assistance Units; **WW = Weekly Workload; ^††^WD = Weekly Days; ^‡‡^TSI = Technical Safety Index; ^§§^NS = Nursing Staff; ^||||^T = Number of ICU Teams (five or four); ^¶¶^COFEN = *Conselho Federal de Enfermagem*

### Ethical aspects

The study was approved by the local Ethics Committee (Certificate of Presentation of Ethical Appreciation: 16288619.0.0000.5327).

## Results

A total of 9,610 NAS evaluations were analyzed, with a mean value of 85.9%. The minimum mean was identified in ICU C in August (80.8%) and the maximum was recorded in ICU D in June (96.4%). The average NAS for each month and the number of evaluations performed in each of the ICUs are shown in Table 1.

When applying Calculation I, an estimated 144 to 179 nursing professionals were identified over the months, of which 75 to 93 should be nurses. When Calculation II was applied, considering the five work shifts present at the institution where the study was conducted and the 15% increase in technical safety, the need for 154 to 192 nursing professionals was estimated, with 80 to 100 nurses. When four work shifts were computed, which are more frequent in Brazilian hospital institutions, the staff was estimated at 123 to 153 nursing professionals, with 64 to 80 nurses ^(Table 2)^.

**Table 1:** - Mean NAS* and number of evaluations performed in the five ICUs^†^ between January and September 2023. Data expressed through the mean NAS* value and absolute number of evaluations in parentheses. Porto Alegre, RS, Brazil, 2023

**Month**	**ICU** ^‡^ **A**	**ICU** ^‡^ **B**	**ICU** ^‡^ **C**	**ICU** ^‡^ **D**	**ICU** ^‡^ **E**	**Total**
January	87.1 (233)	81.2 (242)	82.7 (229)	85.7 (243)	87.1 (121)	84.5 (1.068)
February	81.2 (188)	84.8 (246)	82.4 (210)	86.2 (208)	84.3 (106)	83.8 (958)
March	83.0 (207)	86.6 (270)	82.9 (245)	87.9 (234)	84.8 (122)	85.2 (1.078)
April	85.6 (215)	83.1 (236)	86.1 (218)	89.3 (239)	90.0 (132)	86.6 (1.040)
May	86.9 (203)	84.7 (299)	88.0 (250)	90.6 (242)	86.8 (132)	87.4 (1.126)
June	85.1 (180)	83.1 (234)	86.4 (240)	96.4 (212)	87.2 (128)	87.6 (994)
July	83.7 (218)	83.4 (280)	87.3 (246)	94.0 (228)	88.9 (120)	87.2 (1.92)
August	83.7 (200)	86.6 (305)	80.8 (259)	91.9 (261)	88.9 (135)	86.3 (1.160)
September	84.8 (227)	81.7 (235)	80.6 (258)	89.3 (236)	88.5 (108)	84.4 (1.094)
Total	84.6 (1,871)	84.0 (2,377)	84.1 (2,155)	90.1 (2,103)	87.5 (1,104)	85.9 (9,610)

*NAS = Nursing Activities Score; ^†^ICUs = Intensive Care Units; ^‡^ICU = Intensive Care Unit

**Table 2:** - NAS* values and other elements for Calculations I and II of the sizing of nursing professionals between January and September 2023. Calculation II (a) refers to five work shifts, while Calculation II (b) deals with four shifts. Porto Alegre, RS, Brazil, 2023

**Month**	NAS*				**Calculation I**			**Calculation II (a) - 5 shifts**			**Calculation II (b) - 4 shifts**		
	**Total score month**	**Total score day**	**Total score day x 14.4**	Mean NAS* (%)	**TNH** ^†^	**S** ^‡^ **= TNH** ^†^ **x MK ^§^ (Total team)**	**Nursing staff**	**Score of the day/100 x 5 teams**	**Plus 15% (Total team)**	**Nursing staff**	**Score of the day/100 x 4 teams**	**Plus 15% (Total team)**	**Nursing staff**
January	90,250	3,008	43,320.00	84.5	722.00	161	84	150	173	90	120	138	72
February	80,289	2,676	38,538.72	83.8	642.31	144	75	134	154	80	107	123	64
March	91,795	3,060	44,061.60	85.2	734.36	164	85	153	176	91	122	141	73
April	90,023	3,001	43,211.04	86.6	720.18	161	84	150	173	90	120	138	72
May	98,367	3,279	47,216.16	87.4	786.94	176	91	164	189	98	131	151	78
June	87,098	2,903	41,807.04	87.6	696.78	156	81	145	167	87	116	134	69
July	95,181	3,173	45,686.88	87.2	761.45	170	89	159	182	95	127	146	76
August	100,068	3,336	48,032.64	86.3	800.85	179	93	167	192	100	133	153	80
September	92,329	3,078	44,317.92	84.4	738.63	165	86	154	177	92	123	142	74
Mean of months	91,711	3,057	44,021.33	85.9	733.68	164	85	153	176	91	122	140	73

*NAS = Nursing Activities Score; ^†^TNH = Total Nursing Hours; ^‡^S = Staff; ^§^MK = 0.2236 (Marinho’s Constant, coefficient deducted from the hours and days worked per week and the Technical Safety Index)

## Discussion

In this study, using the NAS as the main calculation component, it was possible to determine the recommended staff for a 55-bed ICC, based on two equations, one of which was further divided into two possibilities, according to the number of work shifts. While Calculation I demonstrated that, on average, 164 professionals were needed, Calculation II, when considering the presence of five or four shifts, determined 176 or 140 workers, respectively.

It was found that, despite the calculation used, over the months, the variation in the estimated staff number was 30 or more professionals. In particular, when using Calculation II for five shifts, the difference between the months of May and August was 38 professionals. Among the 9,610 NAS evaluations performed in the five ICUs of the study, the average NAS was 85.9%, corresponding to 20.2 hours of nursing care/patient in the 24 hours of the day, a number higher than other Brazilian studies ^(26-27)^ and also from countries such as Italy ^(28)^, Portugal ^(10)^ and New Zealand ^(29)^. This daily workload estimate also differs from the current national regulations on sizing the nursing staff, which parameterizes 18 hours of care/day for patients considered to require intensive care ^(19)^. Such discrepancies can be explained by the heterogeneity of ICUs and the characteristics of hospitalized patients.

It has been reported that critically ill patients admitted to public hospitals have a higher mean admission NAS (68.1%) compared to those admitted to private hospitals (56%) ^(30)^. The ICC in this study predominantly serves complex patients from the *Sistema Único de Saúde*. Furthermore, the place is a regional reference center for several specialties, including transplants, which may explain the higher NAS value in relation to other Brazilian public hospitals.

In fact, the total NAS value found in this study varied over the months, as the highest mean score was observed in the month of May (98,367), while the lowest score was found in the months of February and August (80,289). Similarly, an Italian historical series that followed 5,856 patients over five years included 28,390 evaluations and identified NAS variation over the semesters, such that the minimum was: 61.0±17.1%; and the maximum: 68.6±17.4% ^(28)^.

Therefore, determining, through robust longitudinal studies, whether the variation documented in this study is due to chance or whether it will repeat itself over the years can contribute greatly to more rational planning of workforce distribution, for example, in the provision of vacations and periods with greater quantitative reinforcement of workers.

The variability in the estimate of nursing staff obtained when applying each of the methods, as well as the two variations of Calculation II for five or four work shifts, cannot be explained by the variation in the NAS, since the NAS score did not differ in the operationalization of the equations. This means that the equation/calculation method for sizing the nursing team in the ICU has a direct impact on the numerical and categorical definition of the composition of the professional staff. In other words, it is not a question of “merely” defining the instrument for measuring the workload, since in both cases this was concentrated on the NAS, but also of observing what data processing will be employed. This is the direct contribution of this study.

The main difference between the two calculations tested in this pioneering study is due to the fact that the first one transformed the unit’s NAS mean into nursing hours, according to the parameters of the original instrument (each point equivalent to 14.4 minutes) ^(21-22)^ and, with this done, it was based on the equation already recommended by COFEN ^(17)^ for hospital units at the time of the study. In turn, Calculation II was based on the recommendation to predict one professional for every 100 NAS points, which is also in line with the original assumptions of the instrument ^(21-22)^. Furthermore, the calculation results depended on the number of nursing teams working in the sized ICU.

Although it is legitimate and necessary to recognize the innovation of the Calculation II method, it is believed that Calculation I is the most appropriate method for three reasons: 1) it is more directly based on the normative assumptions of the class entity in the country; 2) it is intermediate, considering the minimum and maximum values found in the research, therefore indicating reasonableness; 3) it does not incur the possibility of establishing the total number of workers that is clearly discrepant when considering the number of work shifts in the equation, disfavoring nursing teams that are divided into four shifts, which is more common in the Brazilian scenario.

Even considering that Calculation I is more appropriate and that, from it, elements standardized by COFEN were used, the methodology proposed in this study is not directly referred to by this body, both in 2017 ^(17)^ and in 2024 ^(19)^, which justifies the need for greater methodological depth on planning nursing staff in intensive care, and that this advances in legal terms. Another point to be assumed is that using methodology I, the hours estimated under contractual conditions will be considered ^(17)^, which (in)directly, but not so incisively, tends to have an impact on the number of teams.

Allocating nursing professionals into shifts is a necessity for organizing uninterrupted hospital work. Regarding this, Calculation II tends to favor teams organized in five shifts when considering the total number of workers sized, as well as the number of nurses. However, if divided between shifts and removing 15% of absence coverage that is not distributed on a work schedule ^(31)^, the total number of nursing professionals per shift (n=30) would be equivalent if the workers were divided into either four or five teams.

The above information is relevant when considering the allocation of the nursing workforce linked to budgetary elements. Evidently, the number of staff to compose four teams is less than five, and, consequently, if an equivalent remuneration incentive were hypothesized, sectors operating with four teams would be more economically interesting for the sustainability of organizations. Despite the possible advantage from a financial perspective, it should be added that ICU nursing workers distributed across five teams compared to four have better working conditions, greater job satisfaction and a lower perception of workload ^(32)^. Such results tend to bring favorable clinical outcomes for patients and can also result in savings for healthcare organizations ^(1)^.

The research ^(23)^ carried out in an ICU in Paraná applied the NAS to 107 patients, obtaining an average of 697.3 points in the eight-bed unit. To determine the proportion of nurses on the team, the authors used the parameter proposed by COFEN at the time of the study. As a result, they found that the number of nursing professionals available was outdated and that the proportion of nurses did not meet the Council’s recommendation (35.7% vs 52.5%) ^(23)^. It is important to highlight that the study cited is the one that developed Calculation II, used in this research, and that the proportion of sized staff at the time was slightly higher than that recommended in the regulations valid at the time of the present study ^(17)^, remaining the same after “updating” in 2024 ^(19)^. This reduction may reflect the resistance of health services to hiring higher-level professionals, which is worrying, since it is up to the professional association to represent its interests, in addition to ensuring coherence with the deontological assumptions of nursing.

For more than three decades, COFEN has defined that nurses are the best qualified professionals to provide highly complex care to patients at risk of death ^(12)^, such as those admitted to the ICU. On the other hand, the same entity establishes that 52% of professionals who provide care in ICU must be nurses ^(19)^. In this sense, it still seems necessary to address the evident gap between these two guidelines, which reinforces the importance of greater methodological depth on the planning of nursing staff in the ICU, configuring a contribution of this study.

In the context of intensive care, the technical regulations that establish criteria for the operation of these services, among others, address the distribution of the nursing workforce ^(11)^. However, it differs from what is recommended in the law on professional practice ^(12)^ and in the category’s regulations on the sizing of nursing staff ^(19)^. This contradiction demands actions to strengthen the legal apparatus and inspection, with regard to the minimum number of nursing workers.

Although it is validated for use in Brazil ^(21)^ and internationally recognized ^(7,9-10)^, the NAS was derived from a different scenario than the national one ^(22)^. Aspects related to the organization of Brazilian nursing work need to be considered, since the NAS refers to “nursing professional” – something common for countries where there is no technical and hierarchical division of the category. A recent systematic review that included 23 observational studies conducted in the United States of America, Australia, Belgium, China, South Korea and the United Kingdom concluded that, despite the methodological bias present in some of the articles included in the study, hiring nurses instead of technical professionals, in addition to adjusting the number of staff, improves clinical outcomes and hospital costs ^(33)^.

The NAS, like other tools developed to measure nursing workload, does not consider variables such as the physical structure of services and the specific work dynamics of each care area (in this case, each ICU), in addition to experience and the level of professional competence. Despite this, the cohort study that compared the nursing workload measured by the NAS in relation to the diagnosis of COVID-19 confirmed that the group of patients who tested positive for the diagnosis had a higher workload ^(8)^, which may be evidence that, although reviews are useful, the NAS remains sensitive to capturing fluctuations in the demand/dependence on care and nursing hours in the ICU. Therefore, it meets the ideals of the instrument’s design ^(21-22)^.

Even if a scale estimates that the degree of dependence on nursing care of two patients allows both to be cared for by a single nursing professional, in practice, this will only be possible if both are in beds side by side, or with a proximity that allows the nursing professional to simultaneously view and monitor both patients. In other words, the distribution of the nursing workforce, in addition to the instrumental apparatus, also requires the experience and common sense of nurses and managers.

The present study aimed to bring to light some discussions about the sizing and composition of nursing staff for the care of critically ill patients in the ICU, appreciating methodological procedures to make these ends possible. Despite the large number of observations, it is limited to a few months of the year and to a single center, which may influence the NAS values and staff number estimates. On the other hand, the research documents the need for studies that contribute to the understanding of the components of the calculation of nursing staff, in order to establish a safe and, at the same time, viable patient-to-professional ratio. Nurses must be protagonists in these and other managerial definitions regarding professional practice. Representative entities of the class have the mission of providing support and assistance for this.

## Conclusion

This study demonstrated the recommended nursing staff sizing for a 45-bed ICC, based on two calculations, one of which was divided into two possibilities, according to the number of work shifts. The NAS was used as one of the central elements in the calculations presented. Its stability reinforced the possibility of using it for this purpose.

It is concluded that the calculation methods demonstrated generate clearly discrepant results, especially considering the total absolute number of professionals (whether at mid-level or higher level) deduced by the equations. When comparing the two calculations, it is believed that Calculation I is more appropriate, since it is not so directly affected by the distribution of work shifts, which is not a constant in the organization of nursing work. Therefore, the current Brazilian regulations valid on the sizing of nursing staff in intensive care lack greater methodological depth on the estimation of nursing staff in the ICU context.
