# Supplementary material for: Demonstration of sizing nursing staff methods in intensive care
Source: Rev Lat Am Enfermagem. 2025 Jan 27;33:e4410. doi: 10.1590/1518-8345.7184.4410 (PMC11774145; doi:10.1590/1518-8345.7184.4410)
Supplement: Translation

# Translation: Demostración de métodos de cálculo para dimensionar el equipo de enfermería en cuidados intensivos

**DOI:** 10.1590/1518-8345.7184.4409

**Authors:** Érica Batassini, João Lucas Campos de Oliveira, Mariur Gomes Beghetto

**Affiliations:** 1 Hospital de Clínicas de Porto Alegre, Serviço de Enfermagem em Terapia Intensiva, Porto Alegre, RS, Brasil.; 2 Universidade Federal do Rio Grande do Sul, Escola de Enfermagem, Porto Alegre, RS, Brasil.

**Keywords:** Reducción de Personal, Carga de Trabajo, Grupo de Enfermería, Unidades de Cuidados Intensivos, Personal de Enfermería en Hospital, Cuidados Críticos

## Abstract

**Objetivo:**

demostrar el dimensionamiento del personal de enfermería en cuidados intensivos estimado mediante dos cálculos, utilizando el Nursing Activities Score como uno de sus componentes centrales.

**Método:**

estudio descriptivo, retrospectivo, que recopiló los puntajes del *Nursing Activities* Score de pacientes de cinco Unidades de Cuidados Intensivos de un hospital del sur de Brasil. Se utilizaron dos cálculos para dimensionar el personal de enfermería. Además de otros componentes, el cálculo I convirtió la puntuación del *Nursing Activities* Score en minutos y horas; y el cálculo II utilizó este puntaje sobre un denominador correspondiente a un profesional de enfermería/día, considerando dos tipos de turnos de trabajo. En ambas ecuaciones se respetó la proporción del 52% de enfermeros.

**Resultados:**

en las 9.610 evaluaciones, la puntuación media del *Nursing Activities* Score fue del 85,9% (80,8% a 96,4%). Mientras el cálculo I estimó 164 profesionales, el cálculo II proyectó 176 y 140 trabajadores para cinco y cuatro turnos, respectivamente. La diferencia de enfermeros entre métodos llegó a 18 profesionales.

**Conclusión:**

la elección del cálculo y el número de turnos de trabajo interfieren sustancialmente en la estimación del personal de enfermería de cuidados intensivos. Esto puede fundamentar una revisión metodológica en la estandarización del dimensionamiento de enfermería en esta área.

## Destacados

**(1)** La proyección del personal de enfermería de cuidados intensivos depende del método de cálculo.

**(2)** Se debe revisar la normativa técnica sobre el personal de enfermería en cuidados intensivos.

**(3)** El instrumento de medición de la carga de trabajo no garantiza una proyección igualitaria del personal.

## Introducción

La evidencia sobre la relación directamente proporcional entre una mayor carga de trabajo de enfermería y peores resultados clínicos y resultados relacionados con la seguridad del paciente es exponencial, incluyendo una mayor probabilidad de mortalidad^(1-2)^. El aumento de la carga de trabajo también repercute en la psicodinámica de los profesionales de enfermería, afectando negativamente a su satisfacción con el trabajo y a la percepción de agotamiento^(3)^. En cuidados intensivos, esta realidad se confirma y puede incluso empeorar^(4-5)^, considerando la mayor gravedad clínica y el mayor riesgo de aparición de infecciones relacionadas con la asistencia sanitaria, lesiones por presión y errores de medicación por exceso de carga de trabajo, como lo demuestra una revisión sistemática^(6)^.

Un estudio iraní^(7)^ dedujo que tanto las características de los pacientes (tipo y duración de la estancia, género e ingreso clínico o quirúrgico) ingresados en Unidades de Cuidados Intensivos (UCI) como las del equipo de enfermería (turno, tipo de UCI, género y número de pacientes atendidos) afectan la carga de trabajo de estos profesionales. Se reitera que la carga de trabajo es un constructo complejo de medir, y el “mero” número de pacientes por profesional puede no ser la medida más confiable, incluso en la UCI^(4-5)^. La pandemia de coronavirus fue un ejemplo de esto^(8)^. Sin embargo, la proporción entre pacientes y trabajadores de enfermería tiende a afectar negativamente la percepción que los trabajadores tienen de su carga de trabajo, también en cuidados intensivos^(9)^.

En Portugal, una encuesta encontró que, en más de un tercio de los días analizados (n=91), la demanda de trabajo requerida para enfermería era incompatible con los recursos humanos disponibles^(10)^. Este desfase también se observa en Brasil, ya que la discrepancia entre las diferentes legislaciones y directrices sobre la práctica de la enfermería en el cuidado de pacientes críticos es un obstáculo importante para el dimensionamiento de personal para las UCIs. En 2012, una Resolución del Gobierno Federal brasileño estableció que las UCIs deben mantener una proporción mínima de un enfermero por cada 10 pacientes y un técnico de enfermería por cada dos pacientes^(11)^. Por otro lado, desde hace muchas décadas, la ley sobre el ejercicio profesional de la enfermería brasileña determina que la atención directa de enfermería a pacientes graves, con riesgo de vida, es exclusiva del enfermero^(12)^. Aún así, el censo realizado por la *Associação de Medicina Intensiva Brasileira* (AMIB)^(13)^ identificó que el número de técnicos de enfermería que trabajan en la UCI es casi el doble que el de enfermeros.

El *Nursing Activities Score* (NAS) está reconocido internacionalmente como un instrumento sensible y válido para medir la carga de trabajo de enfermería en UCI^(4-10,14)^. El NAS se considera mejor para medir la carga de trabajo en comparación con el *Therapeutic Intervention Scoring System-28* (TISS-28) y el *Nine Equivalents of Nursing Manpower Use Score* (NEMS)^(15)^. Pese a ello, la medición sistemática y validada de la carga de trabajo no garantiza, por sí sola, la proyección/dimensionamiento del personal de enfermería compatible con la realidad de la demanda laboral, ya que el dimensionamiento del personal de enfermería, aunque dependiente, es un paso posterior de la medición de la carga de trabajo^(16-17)^.

Una revisión de alcance^(18)^ dirigida por reconocidos investigadores ingleses en el área de la planificación del personal de enfermería recomienda que, a pesar del gran volumen de publicaciones, la evidencia sobre los métodos de proyección de enfermeros sigue siendo muy limitada. Los autores señalan, además, que no hay evidencia que respalde la elección de ningún instrumento en particular y recomiendan que las investigaciones futuras se centren en aprender más sobre el uso de los existentes, en lugar de simplemente desarrollar otros nuevos^(18)^.

En Brasil, la normativa del *Conselho Federal de Enfermagem* (COFEN) sobre los parámetros de planificación y dimensionamiento del personal, luego de exigencias legales, pasó de una resolución^(17)^ a un dictamen técnico^(19)^, que, a pesar de incluir un método de dimensionamiento para unidades de atención ininterrumpida, como las UCIs, no aborda metodológicamente la especificidad de estos peculiares espacios de atención. Las variaciones en la medición de la carga de trabajo de enfermería en cuidados intensivos y, en consecuencia, en la proyección de personal, son un obstáculo para establecer la relación de esa variable con los resultados clínicos^(20)^, que se sabe son valiosos para mejorar los cuidados críticos y también fortalecer las justificaciones para la adecuación de recursos humanos.

Existe dificultad para predecir asertivamente cuántos y cuáles profesionales de enfermería se necesitan para atender al paciente crítico^(5)^, lo que va en línea con el pensamiento de que los métodos y estrategias de previsión de los recursos humanos de enfermería merecen ser revisados continuamente^(16-18)^. Admitiendo que la previsión de recursos humanos de enfermería en la UCI puede comprometer la calidad de la atención, pero también perjudicar la sostenibilidad organizacional, el objetivo de este estudio fue demostrar el dimensionamiento del personal de enfermería en cuidados intensivos estimado mediante dos cálculos, utilizando el NAS como uno de sus componentes centrales.

## Método

### Diseño del estudio

Estudio descriptivo, cuantitativo y retrospectivo. Se caracterizó por el análisis de datos primarios obtenidos por medio del sistema computarizado de la institución de investigación.

### Lugar

El estudio se llevó a cabo en un Centro de Cuidados Intensivos (CCI) general (excepto trauma) de un hospital público universitario con capacidad extra en el sur de Brasil. El CCI cuenta con 45 camas distribuidas en cinco UCIs que reciben adultos con diferentes características, a saber: UCI “A”: pacientes clínicos con trastornos cardiovasculares y/o postoperatorios de cirugía cardíaca, con 10 camas; UCI “B”: pacientes quirúrgicos, con 10 camas, y UCIs “C”, “D” y “E”, que atienden a pacientes clínicos de diferentes especialidades, siendo las unidades C y D con 10 camas cada una, y E con 5 camas. No hubo cambios en el número de camas durante el período de estudio.

El equipo de enfermería del CCI trabaja, mayoritariamente, 36 horas semanales. El personal está distribuido de la siguiente manera: las UCIs A, B, C y D cuentan con 10 enfermeros y 39 técnicos de enfermería cada una, distribuidos en cinco turnos de trabajo. En la UCI E hay cinco enfermeros y 22 técnicos de enfermería para los cinco turnos. En el personal general del CCI hay dos enfermeros de apoyo a hemodiálisis y 14 enfermeros interinos. También se cuenta con cinco técnicos de enfermería de apoyo (uno por cada turno). No hay técnicos de enfermería interinos.

### Período

La investigación abarcó el período de enero a septiembre de 2023, tiempo suficiente para dimensionar las unidades hospitalarias^(17,19)^. Además, el período se definió posterior a la declaración del fin de la emergencia pública relacionada con la COVID-19, ya que el escenario del estudio fue referencia para la atención de pacientes con esa condición.

### Participantes

Se incluyeron todos los valores del NAS (n=9.610) computados durante el período del estudio, sin criterios de exclusión, obtenidos de forma retrospectiva, por medio del sistema computarizado de la institución.

### Variables del estudio

La principal variable del estudio fue la puntuación media del NAS. La aplicación del NAS es realizada por enfermeros del CCI, durante sus rutinas de cuidado, según lo recomendado por el instrumento original traducido y validado para Brasil^(21)^. El NAS^(21)^ se aplica una vez al día, así como en el momento de la salida del paciente (alta u óbito), considerando eventos ocurridos en las últimas 24 horas. Los pacientes que fallecen o son dados de alta del CCI antes de las 24 horas de la hospitalización no tienen puntuación del NAS. Se evaluaron los 23 ítems, subdivididos en siete dominios (actividades básicas, soporte ventilatorio, soporte cardiovascular, soporte renal, soporte neurológico, soporte metabólico e intervenciones específicas) del NAS. A cada ítem se le asignó una puntuación específica, siendo la suma máxima posible de todos los ítems 176,8%^(21-22)^. Luego de la suma, se clasifica la carga de trabajo, de manera que el porcentaje igual al 100% del NAS equivale a la demanda de tiempo de un profesional de enfermería las 24 horas del día para cuidar a un paciente; 50 puntos equivalen al 50% del tiempo de un profesional, y así sucesivamente^(22)^.

### Instrumentos utilizados para recolectar datos e información

Después de aplicar el NAS, los enfermeros registraron los resultados en el sistema electrónico de gestión de pacientes del hospital. Para el presente estudio, los datos se obtuvieron de forma agregada, estratificados por UCI (A, B, C, D y E) y por mes (enero a septiembre de 2023). Se utilizó el sistema de métricas y análisis de datos corporativos (*Business Analytics Strategic Intelligence* - BASE^®^) para generar tablas dinámicas con los datos, exportadas a una hoja de cálculo electrónica (*Excel* ^®^). No se recolectaron datos individuales o que permitieran la identificación de pacientes o trabajadores de enfermería.

### Procesamiento y análisis de los datos

Con los datos de puntuación del NAS en la mano, se realizaron dos cálculos de dimensionamiento del personal, ahora denominados Cálculo I y II, cuyos detalles se describen en la Figura 1. Además, a partir del Cálculo II se produjeron dos alternativas de proyección profesional: a) considerando cinco equipos/turnos de trabajo (mañana, tarde y tres turnos nocturnos) y b) considerando cuatro equipos/turnos de trabajo (mañana, tarde y dos turnos nocturnos). Esta definición metodológica se dio porque el campo de estudio cumple con la primera posibilidad (cinco equipos de trabajo); sin embargo, es ampliamente conocido que la realidad masiva de la enfermería hospitalaria brasileña opera con cuatro turnos de trabajo. En ambos cálculos el Índice de Seguridad Técnica (IST) considerado fue el mínimo del 15%, tal y como se recomienda^(16,19)^.

**Figura 1:** - Elementos de los Cálculos I y II para dimensionar el personal de enfermería en UCI*, utilizando el NAS^†^ como uno de los componentes. Porto Alegre, RS, Brasil, 2023

**Elementos**	**Cálculo I**	**Cálculo II**
Base para medir la carga de trabajo de enfermería y justificación para dimensionar el personal de enfermería en la UCI*	NAS^†(21)^ promedio de la unidad en el período	NAS^†(21)^ promedio de la unidad en el período
Ecuación para dimensionar el personal de enfermería de la UCI*	**P** ^‡^ **= HTE** ^§^ **x KM** ^||^_(UAI_^¶^_)_ Dónde: P^‡^: Personal; HTE^§^: Horas Totales de Enfermería, obtenidas de la siguiente ecuación: Puntos del NAS^†^ totales en el día x 14,4 minutos/60 KM^||^: Constante de Marinho, coeficiente deducido de las horas (CHS**) y días semanales (DS^††^) trabajados (siete) y el Índice de Seguridad Técnica (IST^‡‡^)^(17)^. En el presente estudio, KM^||^ = 0,2236, compatible con la jornada laboral de 36 horas semanales y un IST mínimo del 15%. KM^||^_(UAI_^¶^_)_ = DS^††^ x (1 + IST^‡‡^)^(17)^.	**PE** ^§§^ **= E** ^||||^ **x (promedio del NAS** ^†^ **/100) + 15%** Dónde: PE^§§^: Personal de enfermería; E^||||^: número de equipos de la UCI* (cinco o cuatro). IST^‡‡^: Índice de Seguridad Técnica. En este cálculo, IST^‡‡^ = 15%.
Fundamento teórico-científico de la ecuación	Ecuación propuesta por COFEN^¶¶(17)^ para unidades de atención ininterrumpida (UAI^¶^), vigente al momento del estudio. Horas de enfermería extraídas de la conversión de la puntuación del NAS^†^ a minutos, donde cada punto equivale a 14,4 minutos^(22)^.	Propuesta de ecuación presentada en un estudio de 2010^(23)^ e investigaciones posteriores que la utilizaron^(24-25)^. El valor de 100 como denominador del promedio del NAS^†^ en la ecuación de cálculo hace referencia al razonamiento de que este equivale a la demanda de tiempo de un profesional de enfermería las 24 horas del día^(22)^.
Porcentaje de enfermeros sobre el total del personal dimensionado	52%, asumiendo que la población atendida en la UCI* son, en su mayoría, pacientes de cuidados intensivos ^(17,19)^.	52%, suponiendo que la población atendida en la UCI* es, en su mayoría, de cuidados intensivos^(17,19)^.

*UCI = Unidad de Cuidados Intensivos; ^†^NAS = *Nursing Activities Score*; ^‡^P = Personal; ^§^HTE = Horas Totales de Enfermería; ^||^KM = Constante de Marinho; ^¶^UAI = Unidades de Atención Ininterrumpida; **CHS = Carga Horaria Semanal; ^††^DS = Días Semanales; ^‡‡^IST = Índice de Seguridad Técnica; ^§§^PE = Personal de Enfermería; ^||||^E = Número de Equipos de la UCI (cinco o cuatro); ^¶¶^COFEN = *Conselho Federal de Enfermagem*

### Aspectos éticos

El estudio fue aprobado por el Comité de Ética local (Certificado de Presentación de Apreciación Ética: 16288619.0.0000.5327).

## Resultados

Se analizaron 9.610 evaluaciones del NAS, con un valor medio del 85,9%. El promedio mínimo se identificó en la UCI C en agosto (80,8%) y el máximo se registró en la UCI D en junio (96,4%). El NAS promedio de cada mes y el número de evaluaciones realizadas en cada una de las UCIs se muestran en la Tabla 1.

Al aplicar el Cálculo I, a lo largo de los meses, se identificó un número estimado de 144 a 179 profesionales de enfermería, de los cuales 75 a 93 deberían ser enfermeros. Cuando se aplicó el Cálculo II, considerando los cinco turnos de trabajo presentes en la institución sede del estudio y el aumento del 15% en la seguridad técnica, se estimó la necesidad de 154 a 192 profesionales de enfermería, es decir, 80 a 100 enfermeros. Cuando se calcularon cuatro turnos de trabajo, más frecuentes en las instituciones hospitalarias brasileñas, se dimensionó el personal entre 123 y 153 profesionales de enfermería, con 64 a 80 enfermeros ^(Tabla 2)^.

**Tabla 1:** - NAS* promedio y número de evaluaciones realizadas en las cinco UCIs^†^ entre enero y septiembre de 2023. Datos expresados como valor del NAS* promedio y número absoluto de evaluaciones entre paréntesis. Porto Alegre, RS, Brasil, 2023

**Mes**	**UCI** ^‡^ **A**	**UCI** ^‡^ **B**	**UCI** ^‡^ **C**	**UCI** ^‡^ **D**	**UCI** ^‡^ **E**	**Total**
Enero	87,1 (233)	81,2 (242)	82,7 (229)	85,7 (243)	87,1 (121)	84,5 (1.068)
Febrero	81,2 (188)	84,8 (246)	82,4 (210)	86,2 (208)	84,3 (106)	83,8 (958)
Marzo	83,0 (207)	86,6 (270)	82,9 (245)	87,9 (234)	84,8 (122)	85,2 (1.078)
Abril	85,6 (215)	83,1 (236)	86,1 (218)	89,3 (239)	90,0 (132)	86,6 (1.040)
Mayo	86,9 (203)	84,7 (299)	88,0 (250)	90,6 (242)	86,8 (132)	87,4 (1.126)
Junio	85,1 (180)	83,1 (234)	86,4 (240)	96,4 (212)	87,2 (128)	87,6 (994)
Julio	83,7 (218)	83,4 (280)	87,3 (246)	94,0 (228)	88,9 (120)	87,2 (1.92)
Agosto	83,7 (200)	86,6 (305)	80,8 (259)	91,9 (261)	88,9 (135)	86,3 (1.160)
Septiembre	84,8 (227)	81,7 (235)	80,6 (258)	89,3 (236)	88,5 (108)	84,4 (1.094)
Total	84,6 (1.871)	84,0 (2.377)	84,1 (2.155)	90,1 (2.103)	87,5 (1.104)	85,9 (9.610)

*NAS = *Nursing Activities Score*; ^†^UCIs = Unidades de Cuidados Intensivos; ^‡^UCI = Unidad de Cuidados Intensivos

**Tabla 2:** - Valores del NAS* y otros elementos para los Cálculos I y II del dimensionamiento de profesionales de enfermería entre enero y septiembre de 2023. El Cálculo II (a) se refiere a cinco turnos de trabajo, mientras que el Cálculo II (b) se ocupa de cuatro turnos. Porto Alegre, RS, Brasil, 2023

**Mes**	NAS*				**Cálculo I**			**Cálculo II (a) - 5 turnos**			**Cálculo II (b) - 4 turnos**		
	**Total puntuación mes**	**Total puntuación día**	**Total puntuación día x 14,4**	NAS* promedio (%)	**H TE** ^†^	**P** ^‡^ **= HTE** ^†^ **x KM** ^§^ **(Equipo total)**	**Personal de enfermería**	**Puntuación del día/100 x 5 equipos**	**Más 15% (Equipo total)**	**Personal de enfermería**	**Puntuación del día/100 x 4 equipos**	**Más 15% (Equipo total)**	**Personal de enfermería**
Enero	90.250	3.008	43.320,00	84,5	722,00	161	84	150	173	90	120	138	72
Febrero	80.289	2.676	38.538,72	83,8	642,31	144	75	134	154	80	107	123	64
Marzo	91.795	3.060	44.061,60	85,2	734,36	164	85	153	176	91	122	141	73
Abril	90.023	3.001	43.211,04	86,6	720,18	161	84	150	173	90	120	138	72
Mayo	98.367	3.279	47.216,16	87,4	786,94	176	91	164	189	98	131	151	78
Junio	87.098	2.903	41.807,04	87,6	696,78	156	81	145	167	87	116	134	69
Julio	95.181	3.173	45.686,88	87,2	761,45	170	89	159	182	95	127	146	76
Agosto	100.068	3.336	48.032,64	86,3	800,85	179	93	167	192	100	133	153	80
Septiembre	92.329	3.078	44.317,92	84,4	738,63	165	86	154	177	92	123	142	74
Promedio mensual	91.711	3.057	44.021,33	85,9	733,68	164	85	153	176	91	122	140	73

*NAS = *Nursing Activities Score*; ^†^HTE = Horas Totales de Enfermería; ^‡^P = Personal; ^§^KM = 0,2236 (Constante de Marinho, coeficiente deducido de las horas y días semanales trabajados y del Índice de Seguridad Técnica)

## Discusión

En el presente estudio, utilizando el NAS como componente principal de los cálculos, fue posible dimensionar el personal recomendado para un CCI de 45 camas, en base a dos ecuaciones, una de las cuales se dividió en dos posibilidades, según el número de turnos de trabajo. Mientras el Cálculo I demostró que, en promedio, se necesitaban 164 profesionales, el Cálculo II, al considerar la presencia de cinco o cuatro turnos, determinó 176 o 140 trabajadores, respectivamente.

Se constató que, a pesar del cálculo utilizado, a lo largo de los meses, la variación en el personal estimada fue de 30 o más profesionales. Especialmente, al utilizar el Cálculo II para cinco turnos, la diferencia entre los meses de mayo y agosto fue de 38 profesionales. Entre las 9.610 evaluaciones del NAS realizadas en las cinco UCI del estudio, el NAS promedio fue del 85,9%, correspondiente a 20,2 horas de atención de enfermería/paciente durante las 24 horas del día, número superior al de otros estudios brasileños^(26-27)^ y también de países como Italia^(28)^, Portugal^(10)^ y Nueva Zelanda^(29)^. Esta estimación de carga de trabajo diaria también difiere de la actual normativa nacional sobre dimensionamiento de enfermería, que fija 18 horas de cuidado/día para los pacientes considerados de cuidados intensivos^(19)^. Estas divergencias pueden explicarse por la heterogeneidad de las UCIs y las características de los pacientes hospitalizados.

Se ha reportado que los pacientes críticos ingresados ​​en hospitales públicos tienen un NAS promedio de ingreso mayor (68,1%) en comparación con los ingresados ​​en hospitales privados (56%)^(30)^. El CCI del presente estudio atiende predominantemente a pacientes complejos del *Sistema Único de Saúde*. Además, el lugar es un centro de referencia regional para varias especialidades, incluidos trasplantes, lo que puede explicar el mayor valor del NAS en relación a otros hospitales públicos brasileños.

De hecho, el valor total del NAS encontrado en este estudio varió a lo largo de los meses, ya que el puntaje promedio más alto se observó en el mes de mayo (98.367), mientras que el puntaje más bajo se encontró en los meses de febrero y agosto (80.289). De manera similar, una serie histórica italiana que siguió a 5.856 pacientes durante cinco años incluyó 28.390 evaluaciones e identificó variación en el NAS a lo largo de los semestres, de modo que el mínimo fue: 61,0 ± 17,1%; y el máximo: 68,6±17,4%^(28)^.

Por lo tanto, determinar, mediante estudios longitudinales sólidos, si la variación documentada en el presente estudio se debe al azar o si se repetirá a lo largo de los años puede contribuir en gran medida a una planificación más racional de la distribución de la fuerza laboral, por ejemplo, en la provisión de vacaciones y períodos con un mayor número de trabajadores.

La variabilidad en la estimación del personal de enfermería obtenida al aplicar cada uno de los métodos, así como las dos variaciones en el Cálculo II para cinco o cuatro turnos de trabajo, no pueden explicarse por la variación en el NAS, ya que la puntuación NAS no difirió en la operacionalización de las ecuaciones. Esto significa que la ecuación/método de cálculo para dimensionar el equipo de enfermería en la UCI tiene un impacto directo en la definición numérica y categórica de la composición del cuadro profesional. Es decir, no se trata “meramente” de definir el instrumento de medición de la carga de trabajo, ya que en ambos casos esta se concentró en el NAS, sino también de observar qué procesamiento de datos se utilizará. Esta es la contribución directa de este estudio.

La principal diferencia entre los dos cálculos pioneros en este estudio se debe a que el primero transformó el NAS promedio de la unidad en horas de enfermería, según los parámetros del instrumento original (cada punto equivalente a 14,4 minutos)^(21-22)^ y, hecho esto, se basó en la ecuación ya recomendada por COFEN^(17)^ para unidades hospitalarias al momento del estudio. A su vez, el Cálculo II se basó en la recomendación de dotar de un profesional por cada 100 puntos del NAS, lo que también concuerda con los supuestos originales del instrumento^(21-22)^. Además, los resultados del cálculo dependieron del número de equipos de enfermería que trabajaban en la UCI dimensionada.

Si bien es legítimo y necesario reconocer la innovación del método del Cálculo II, se cree que el Cálculo I es el método más apropiado por tres razones: 1) se basa más directamente en los supuestos normativos de la entidad de clase en el país; 2) es intermedio, considerando los valores mínimo y máximo encontrados en la investigación, por lo que indica razonabilidad; 3) no incurre en la posibilidad de establecer el número total de trabajadores claramente discrepante al considerar el número de turnos de trabajo en la ecuación, desfavoreciendo a los equipos de enfermería que se dividen en cuatro turnos, lo que es más común en el escenario brasileño.

Aun considerando que el Cálculo I es más adecuado, y que a partir de él se utilizaron elementos estandarizados por el COFEN, la metodología propuesta en este estudio no es referida directamente por este organismo, tanto en 2017^(17)^ como en 2024^(19)^, lo que justifica la necesidad de profundizar metodológicamente en la planificación del personal de enfermería en cuidados intensivos, y que esto avance en términos jurídicos. Otro punto a asumir es que utilizando la metodología I se considerarán las horas previstas bajo un régimen contractual^(17)^, lo que (in)directamente, pero no de manera tan incisiva, tiende a impactar en el número de equipos.

La asignación de turnos a los profesionales de enfermería es una necesidad para organizar el trabajo hospitalario ininterrumpido. En este sentido, el Cálculo II tiende a favorecer equipos organizados en cinco turnos al considerar el número total de trabajadores dimensionados, así como el número de enfermeros. Sin embargo, si se dividiera entre turnos y se eliminara el 15% de la cobertura de ausencias que no se distribuyen en horario de trabajo^(31)^, el número total de profesionales de enfermería por turno (n=30) sería equivalente si los trabajadores se dividieran en cuatro o cinco equipos.

La información anterior es relevante cuando se piensa en la asignación de la fuerza laboral de enfermería vinculada a elementos presupuestarios. Evidentemente, el número de personal para formar cuatro equipos es inferior a cinco y, en consecuencia, si se plantea la hipótesis de un incentivo retributivo equivalente, sectores que operen con cuatro equipos serían económicamente más interesantes para la sostenibilidad de las organizaciones. A pesar de la posible ventaja desde el punto de vista económico, se añade que los trabajadores de enfermería de UCI distribuidos en cinco equipos frente a cuatro tienen mejores condiciones laborales, mayor satisfacción con el trabajo y menor percepción de carga de trabajo^(32)^. Estos resultados tienden a generar resultados clínicos favorables para los pacientes y también pueden generar ahorros para las organizaciones de salud^(1)^.

La investigación^(23)^ realizada en una UCI de Paraná aplicó el NAS a 107 pacientes, obteniendo un promedio de 697,3 puntos en la unidad de ocho camas. Para determinar la proporción de enfermeros en el equipo, las autoras utilizaron el parámetro propuesto por COFEN en el momento del estudio. Como resultado, encontraron que el número de profesionales de enfermería disponibles estaba desactualizado y que la proporción de enfermeros no cumplía con la recomendación del Consejo (35,7% frente a 52,5%)^(23)^. Es importante resaltar que el estudio citado es el que desarrolló el Cálculo II, utilizado en la presente investigación, y que la proporción de enfermeros en el personal dimensionado en su momento fue levemente superior a lo recomendado en la normativa vigente en el momento del presente estudio^(17)^, y permaneció después de la “actualización” en 2024^(19)^. Esta reducción puede reflejar la resistencia de los servicios de salud a contratar profesionales de educación superior, lo cual es preocupante, ya que corresponde a la entidad profesional representar los intereses de la profesión, además de garantizar la coherencia con los supuestos deontológicos de la enfermería.

Desde hace más de tres décadas, COFEN ha definido que los enfermeros son los profesionales mejor calificados para brindar cuidados de alta complejidad a pacientes en riesgo de muerte^(12)^, como los ingresados en UCI. Por otro lado, la misma entidad establece que el 52% de los profesionales que brindan atención en UCI deben ser enfermeros^(19)^. En este sentido, todavía parece necesario abordar la brecha evidente entre estas dos guías, lo que refuerza la importancia de una mayor profundidad metodológica en la planificación del personal de enfermería en UCI, configurando una contribución de este estudio.

En el ámbito de los cuidados intensivos, la normativa técnica que establece criterios de funcionamiento de estos servicios, entre otros, aborda la distribución de la fuerza laboral de enfermería^(11)^. Sin embargo, difiere de lo recomendado en la ley de ejercicio profesional^(12)^ y en el reglamento de la categoría sobre dimensionamiento del personal de enfermería^(19)^. Esta contradicción exige acciones para fortalecer el aparato legal y de inspección, en lo que respecta al número mínimo de trabajadores de enfermería.

Aunque está validado para su uso en Brasil^(21)^ y reconocido internacionalmente^(7,9-10)^, el NAS surgió de un escenario diferente al nacional^(22)^. Es necesario considerar aspectos relacionados con la organización del trabajo de enfermería brasileño, ya que el NAS se refiere a “profesional de enfermería” – algo habitual en países donde no existe una división técnica y jerárquica de la categoría. Una revisión sistemática reciente que incluyó 23 estudios observacionales realizados en Estados Unidos de América, Australia, Bélgica, China, Corea del Sur y Reino Unido concluyó que, a pesar del sesgo metodológico presente en algunos de los artículos incluidos en el estudio, la contratación de enfermeros en lugar de profesionales técnicos, además de adaptar el número de personal, mejora los resultados clínicos y los costes hospitalarios^(33)^.

El NAS, así como otras herramientas desarrolladas para medir la carga de trabajo de enfermería, no considera variables como la estructura física de los servicios y la dinámica de trabajo específica de cada área de atención (en este caso, cada UCI), además de la experiencia y el nivel de competencia profesional. Pese a ello, el estudio de cohorte que comparó la carga de trabajo de enfermería medida por el NAS en relación al diagnóstico de COVID-19 confirmó que el grupo de pacientes positivos al diagnóstico tuvo mayor carga de trabajo^(8)^, lo que podría ser una evidencia de que, aunque las revisiones son útiles, el NAS sigue siendo sensible a capturar las fluctuaciones en la demanda/dependencia de la atención y las horas de enfermería en la UCI. Por lo tanto, cumple con los ideales del diseño del instrumento^(21-22)^.

Si bien una escala estima que el grado de dependencia de los cuidados de enfermería de dos pacientes hace posible que ambos sean atendidos por un único profesional de enfermería, en la práctica esto sólo será posible si ambos están en camas uno al lado del otro, o con una proximidad que permite al profesional de enfermería visualizar y monitorear a ambos pacientes simultáneamente. En otras palabras, la distribución de la fuerza laboral de enfermería, además del aparato instrumental, también requiere la experiencia y el sentido común de enfermeros y gestores.

El presente estudio tuvo como objetivo sacar a la luz algunas discusiones sobre el dimensionamiento y composición del personal de enfermería para el cuidado de pacientes críticos en UCI, apreciando procedimientos metodológicos que permitan estos propósitos. A pesar del gran número de observaciones, se limita a unos pocos meses del año y en un solo centro, lo que puede influir en los valores del NAS y en las estimaciones de dotación. Por otro lado, la investigación documenta la necesidad de estudios que contribuyan a la comprensión de los componentes del cálculo del personal de enfermería, con el fin de establecer una relación segura y, al mismo tiempo, viable entre pacientes y profesionales. Los enfermeros deben ser protagonistas de éstas y otras definiciones gerenciales de la práctica profesional. Las entidades representativas de la clase tienen la misión de brindar apoyo y contención para ello.

## Conclusión

El presente estudio demostró el dimensionamiento recomendado del personal de enfermería para un CCI de 45 camas, a partir de dos cálculos, uno de los cuales se dividió en dos posibilidades según el número de turnos de trabajo. El NAS se utilizó como uno de los elementos centrales en los cálculos presentados. Su estabilidad reforzó la posibilidad de utilizarlo para este fin.

Se concluye que los métodos de cálculo demostrados generan resultados claramente discrepantes, especialmente considerando el número absoluto total de profesionales (ya sea de educación secundaria o superior) deducido por las ecuaciones. Al comparar los dos cálculos, se cree que el Cálculo I es más apropiado, ya que no se ve tan directamente afectado por la distribución de los turnos de trabajo, que no es una constante en la organización del trabajo de enfermería. Por lo tanto, la actual normativa brasileña sobre dimensionamiento del personal de enfermería en cuidados intensivos requiere mayor profundidad metodológica sobre la provisión de personal de enfermería en el contexto de UCI.

# Translation: Demonstração de métodos de cálculo para o dimensionamento da equipe de enfermagem em terapia intensiva

**DOI:** 10.1590/1518-8345.7184.4411

**Authors:** Érica Batassini, João Lucas Campos de Oliveira, Mariur Gomes Beghetto

**Affiliations:** 1 Hospital de Clínicas de Porto Alegre, Serviço de Enfermagem em Terapia Intensiva, Porto Alegre, RS, Brasil.; 2 Universidade Federal do Rio Grande do Sul, Escola de Enfermagem, Porto Alegre, RS, Brasil.

**Keywords:** Dimensionamento de Pessoal, Carga de Trabalho, Equipe de Enfermagem, Unidades de Terapia Intensiva, Recursos Humanos de Enfermagem no Hospital, Cuidados Críticos

## Abstract

**Objetivo:**

demonstrar o dimensionamento do pessoal de enfermagem em terapia intensiva estimado por dois cálculos, utilizando o *Nursing Activities Score* como um dos seus componentes centrais.

**Método:**

estudo descritivo, retrospectivo, que compilou as pontuações do *Nursing Activities* Score de pacientes de cinco Unidades de Terapia Intensiva de um hospital do sul do Brasil. Dois cálculos foram empregados para dimensionar o quadro de enfermagem. Além de outros componentes, o cálculo I converteu o escore do *Nursing Activities Score* em minutos e horas; e o cálculo II utilizou este escore sobre um denominador correspondente a um profissional de enfermagem/dia, tendo em vista duas modalidades de turnos de trabalho. Em ambas as equações, respeitou-se a proporção de 52% de enfermeiros.

**Resultados:**

nas 9.610 avaliações, a média do *Nursing Activities Score* foi de 85,9% (80,8% a 96,4%). Enquanto o cálculo I estimou 164 profissionais, o cálculo II projetou 176 e 140 trabalhadores para cinco e quatro turnos, respectivamente. A diferença de enfermeiros entre métodos chegou a 18 profissionais.

**Conclusão:**

a escolha do cálculo e o número de turnos de trabalho interferem substancialmente na estimativa de pessoal de enfermagem intensivista. Isso pode fundamentar revisão metodológica na normatização do dimensionamento de enfermagem desta área.

## Destaques

**(1)** A projeção de pessoal de enfermagem intensivista depende do método de cálculo.

**(2)** A normativa técnica sobre pessoal de enfermagem em terapia intensiva merece revisão.

**(3)** O instrumento de aferição de carga de trabalho não garante projeção de pessoal igual.

## Introdução

As evidências sobre a relação diretamente proporcional do aumento da carga de trabalho de enfermagem com piores desfechos clínicos e resultados relacionados à segurança do paciente são exponenciais, incluindo a maior chance de mortalidade^(1-2)^. A elevação da carga de trabalho também repercute na psicodinâmica do profissional de enfermagem, afetando negativamente sua satisfação com o trabalho e percepção de exaustão^(3)^. Na terapia intensiva, essa realidade se confirma e pode até se agravar^(4-5)^, considerando a maior gravidade clínica e o maior risco de ocorrência de infecções relacionadas à assistência à saúde, lesões por pressão e erros de medicação pela carga de trabalho excessiva, conforme atestou revisão sistemática^(6)^.

Um estudo iraniano^(7)^ inferiu que tanto as características dos pacientes (tipo e tempo de internação, sexo e admissão clínica ou cirúrgica) internados em Unidades de Terapia Intensiva (UTI) como as da equipe de enfermagem (turno, tipo de UTI, sexo e número de paciente sob cuidados) afetam a carga de trabalho destes profissionais. Reitera-se que a carga de trabalho é um construto complexo de ser aferido, e o “mero” número de pacientes por profissional talvez não seja a medida mais confiável para sua mensuração, inclusive em UTI^(4-5)^. A pandemia causada pelo coronavírus foi um exemplo disso^(8)^. Todavia, a proporção de pacientes por trabalhadores de enfermagem tende a afetar negativamente a percepção dos trabalhadores quanto à sua carga de trabalho, inclusive na terapia intensiva^(9)^.

Em Portugal, uma pesquisa verificou que, em mais de um terço dos dias analisados (n=91), a demanda de trabalho requerida à enfermagem foi incompatível com os recursos humanos disponíveis^(10)^. Tal desajuste também é verificado no Brasil, visto que o desencontro entre as diferentes legislações e diretrizes referentes ao exercício da enfermagem no cuidado ao paciente crítico configura-se como um grande dificultador para o dimensionamento do pessoal para as UTIs. Em 2012, uma Resolução do Governo Federal brasileiro estabeleceu que as UTIs devem manter uma razão mínima de um enfermeiro para cada 10 pacientes e de um técnico de enfermagem para cada dois pacientes^(11)^. Por outro lado, há muitas décadas, a lei do exercício profissional da enfermagem brasileira determina que o cuidado de enfermagem direto a pacientes graves, com risco à vida, é privativo do enfermeiro^(12)^. Ainda assim, o censo conduzido pela Associação de Medicina Intensiva Brasileira (AMIB)^(13)^ identificou que o quantitativo de técnicos de enfermagem atuando nas UTI é quase o dobro do que o de enfermeiros.

O *Nursing Activities Score* (NAS) é internacionalmente reconhecido como um instrumento sensível e válido para aferir a carga de trabalho de enfermagem em UTI^(4-10,14)^. O NAS é considerado melhor para aferir a carga de trabalho em comparação ao *Therapeutic Intervention Scoring System-28* (TISS-28) e ao *Nine Equivalents of Nursing Manpower Use Score* (NEMS)^(15)^. Apesar disso, a mensuração sistemática e validada da carga de trabalho não garante, por si só, a projeção/dimensionamento de pessoal de enfermagem compatível com a realidade de demanda laboral, visto que o dimensionamento do pessoal de enfermagem, ainda que dependente, é uma etapa subsequente da medida de carga de trabalho^(16-17)^.

Uma revisão de escopo^(18)^ liderada por pesquisadores ingleses renomados na área de planejamento de pessoal de enfermagem recomenda que, apesar do grande volume de publicações, as evidências sobre os métodos de projeção de enfermeiros permanecem altamente limitadas. Os autores referem ainda que não há evidências para apoiar a escolha de qualquer instrumento em particular e recomendam que pesquisas futuras se concentrem em aprender mais sobre o uso daqueles existentes, ao invés de simplesmente desenvolver novos^(18)^.

No Brasil, a normativa do Conselho Federal de Enfermagem (COFEN) sobre os parâmetros do planejamento e dimensionamento de pessoal, após demandas judiciais, passou de uma resolução^(17)^ para parecer técnico^(19)^, o qual, apesar de incluir método de dimensionamento para unidades de cuidado ininterrupto, como as UTIs, não aborda metodologicamente a especificidade desses espaços assistenciais peculiares. Variações na medida de carga de trabalho de enfermagem intensivista e, consequentemente, na projeção de pessoal são um entrave para estabelecer a relação dessa variável com desfechos clínicos^(20)^, que sabidamente são valiosos para melhorar o cuidado crítico e também fortalecer justificativas de adequação de recursos humanos.

Há dificuldade em predizer com assertividade quantos e quais profissionais de enfermagem são necessários para assistir ao paciente crítico^(5)^, o que coaduna com o pensamento de que os métodos e estratégias de previsão de recursos humanos de enfermagem merecem ser continuamente revisados^(16-18)^. Admitindo que a previsão de recursos humanos de enfermagem em UTI pode comprometer a qualidade do cuidado, mas também onerar a sustentabilidade organizacional, o objetivo deste estudo consistiu em demonstrar o dimensionamento do pessoal de enfermagem em terapia intensiva estimado por dois cálculos, utilizando NAS como um dos seus componentes centrais.

## Método

### Delineamento do estudo

Estudo descritivo, quantitativo e retrospectivo. Caracterizou-se pela análise de dados primários obtidos por meio do sistema informatizado da instituição de inquérito.

### Local

O estudo foi realizado em um Centro de Terapia Intensiva (CTI) geral (exceto trauma) de um hospital público, universitário, de porte extra, no Sul do Brasil. O CTI conta com 45 leitos distribuídos em cinco UTIs que recebem adultos com diferentes características, a saber: UTI “A”: clínicos com distúrbios cardiovasculares e/ou em pós-operatório de cirurgias cardíacas, com 10 leitos; UTI “B”: pacientes cirúrgicos, com 10 leitos, e UTIs “C”, “D” e “E”, que atendem pacientes clínicos de diferentes especialidades, sendo as unidades C e D com 10 leitos cada uma, e a E com 5 leitos. Não houve modificações no quantitativo de leitos no período de estudo.

A equipe de enfermagem do CTI cumpre, majoritariamente, um regime laboral de 36 horas semanais. O quadro de pessoal é distribuído da seguinte forma: as UTIs A, B, C e D contam com 10 enfermeiros e 39 técnicos de enfermagem cada, distribuídos em cinco turnos de trabalho. Na UTI E, são cinco enfermeiros e 22 técnicos de enfermagem para os cinco turnos. No quadro geral do CTI, há dois enfermeiros de apoio para hemodiálise e 14 enfermeiros folguistas. Também há cinco técnicos de enfermagem de apoio (um para cada turno). Não há técnicos de enfermagem folguistas.

### Período

A pesquisa abrangeu o período de janeiro a setembro de 2023, tempo suficiente para dimensionar unidades hospitalares^(17,19)^. Adicionalmente, o período foi definido após a declaração do fim da emergência pública relacionada à COVID-19, uma vez que o cenário do estudo foi referência para atender a pacientes com esse agravo.

### Participantes

Foram incluídos todos os valores de NAS (n=9.610) computados durante o período do estudo, sem critérios de exclusão, obtidos retrospectivamente, por meio do sistema informatizado da instituição.

### Variáveis do estudo

A principal variável do estudo foi o escore médio do NAS. A aplicação do NAS é procedida pelos enfermeiros do CTI, durante suas rotinas assistenciais, conforme a recomendação do instrumento original traduzido e validado para o Brasil^(21)^. O NAS^(21)^ é aplicado uma vez ao dia, bem como no momento da saída do paciente (alta ou óbito), considerando os eventos ocorridos nas últimas 24 horas. Pacientes que evoluem para óbito ou têm alta do CTI antes de 24 horas de internação não têm o NAS pontuado. Foram avaliados os 23 itens, subdivididos em sete domínios (atividades básicas, suporte ventilatório, suporte cardiovascular, suporte renal, suporte neurológico, suporte metabólico e intervenções específicas) do NAS. Para cada item, atribuiu-se uma pontuação específica, sendo que a soma máxima possível de todos os itens foi 176,8%^(21-22)^. Após a soma, a carga de trabalho é classificada, de modo que a porcentagem igual a 100% de NAS equivale à demanda do tempo de um profissional de enfermagem nas 24 horas do dia, para o cuidado a um paciente; 50 pontos equivalem a 50% do tempo de um profissional e assim sucessivamente^(22)^.

### Instrumentos utilizados para a coleta de dados e informações

Após a aplicação do NAS, os enfermeiros registraram os resultados no sistema eletrônico de gerenciamento de pacientes do hospital. Para o presente estudo, os dados foram obtidos de modo agregado, estratificados por UTI (A, B, C, D e E) e por mês (janeiro a setembro de 2023). Foi utilizado o sistema de métricas e análise de dados corporativos (*Business Analytics Strategic Intelligence* - BASE^®^) para gerar as tabelas dinâmicas com os dados, exportadas para uma planilha eletrônica (*Excel* ^®^). Não foram coletados dados individuais ou que permitissem a identificação de pacientes ou de trabalhadores de enfermagem.

### Tratamento e análise dos dados

De posse dos dados de escores do NAS, procedeu-se dois cálculos de dimensionamento de pessoal, ora denominados Cálculo I e II, cujo detalhamento está descrito na Figura 1. Ainda, a partir do Cálculo II, duas alternativas de projeção de profissionais foram produzidas: a) considerando cinco equipes/turnos de trabalho (manhã, tarde e três turnos noturnos) e b) considerando quatro equipes/turnos de trabalho (manhã, tarde e dois turnos noturnos). Tal definição metodológica se deu porque o campo de estudo atende à primeira possibilidade (cinco equipes de trabalho); contudo, é de amplo conhecimento que a massiva realidade da enfermagem hospitalar brasileira atua com quatro turnos de trabalho. Em ambos os cálculos, o Índice de Segurança Técnica (IST) considerado foi o mínimo de 15%, conforme recomendação^(16,19)^.

**Figura 1:** - Elementos dos Cálculos I e II para o dimensionamento de pessoal de enfermagem em UTI*, utilizando o NAS^†^ como um dos componentes. Porto Alegre, RS, Brasil, 2023

**Elementos**	**Cálculo I**	**Cálculo II**
Base para mensuração da carga de trabalho de enfermagem e fundamentação do dimensionamento de pessoal de enfermagem na UTI*	NAS^†(21)^ médio da unidade no período	NAS^†(21)^ médio da unidade no período
Equação para o dimensionamento do quadro de pessoal de enfermagem da UTI*	**QP** ^‡^ **= THE** ^§^ **x KM** ^||^_(UAI_^¶^~) ~ Onde: QP^‡^: Quadro de pessoal; THE^§^: Total de Horas de Enfermagem, obtido a partir da seguinte equação: Total de pontos do NAS^†^ no dia x 14,4 minutos/60 KM^||^: Constante de Marinho, coeficiente deduzido sobre as horas (CHS**) e os dias semanais (DS^††^) trabalhados (sete) e o Índice de Segurança Técnica (IST^‡‡^)^(17)^. No presente estudo, KM^||^ = 0,2236, compatível com a jornada de trabalho de 36 horas semanais e IST mínimo de 15%. KM^||^_(UAI_^¶^_)_ = DS^††^ x (1 + IST^‡‡^)^(17)^.	**PE** ^§§^ **= E** ^||||^ **x (média do NAS** ^†^ **/100) + 15%** Onde: PE^§§^: Pessoal de enfermagem; E^||||^: número de equipes da UTI* (cinco ou quatro). IST^‡‡^: Índice de Segurança Técnica. Neste cálculo, IST^‡‡^ = 15%.
Fundamentação teórico-científica da equação	Equação proposta pelo COFEN^(17)^ para unidades de assistência ininterrupta (UAI), vigente à época do estudo. Horas de enfermagem extraídas da conversão do escore do NAS^†^ em minutos, onde cada ponto equivale a 14,4 minutos^(22)^.	Proposta da equação apresentada em estudo de 2010^(23)^ e pesquisas posteriores que a empregaram^(24-25)^. O valor de 100 como denominador da média do NAS na equação do cálculo refere-se à fundamentação de que isso equivale à demanda do tempo de um profissional de enfermagem nas 24 horas do dia^(22)^.
Percentual de enfermeiros do total de pessoal dimensionado	52%, partindo do pressuposto de que a clientela atendida na UTI* é majoritariamente correspondente aos pacientes intensivos^(17,19)^.	52%, partindo do pressuposto de que a clientela atendida na UTI* é majoritariamente correspondente aos cuidados intensivos ^(17,19)^.

*UTI = Unidade de Terapia Intensiva; ^†^NAS = *Nursing Activities Score*; ^‡^QP = Quadro de Pessoal; ^§^THE = Total de Horas de Enfermagem; ^||^KM = Constante de Marinho; ^¶^UAI = Unidades de Assistência Ininterrupta; **CHS = Carga Horária Semanal; ^††^DS = Dias Semanais; ^‡‡^IST = Índice de Segurança Técnica; ^§§^PE = Pessoal de Enfermagem; ^||||^E = Número de Equipes da UTI (cinco ou quatro); ^¶¶^COFEN = Conselho Federal de Enfermagem

### Aspectos éticos

O estudo foi aprovado pelo Comitê de Ética local (Certificado de Apresentação de Apreciação Ética: 16288619.0.0000.5327).

## Resultados

Foram analisadas 9.610 avaliações de NAS, cujo valor médio foi de 85,9%. A média mínima foi identificada na UTI C em agosto (80,8%) e a máxima foi registrada na UTI D em junho (96,4%). O NAS médio de cada mês e o número de avaliações realizadas em cada uma das UTIs está demonstrado na Tabela 1.

Ao aplicar o Cálculo I, identificou-se, ao longo dos meses, a estimativa de um quadro de 144 a 179 profissionais de enfermagem, dos quais 75 a 93 deveriam ser enfermeiros. Quando aplicado o Cálculo II, considerando-se os cinco turnos de trabalho presentes na instituição sede do estudo e o acréscimo de 15% de segurança técnica, foi estimada a necessidade de 154 a 192 profissionais de enfermagem, sendo 80 a 100 enfermeiros. Quando computados quatro turnos de trabalho, mais frequentes em instituições hospitalares brasileiras, foi dimensionado o quadro de 123 a 153 profissionais de enfermagem, sendo 64 a 80 enfermeiros ^(Tabela 2)^.

**Tabela 1:** - NAS* médio e número de avaliações realizadas nas cinco UTIs^†^ entre janeiro e setembro de 2023. Dados expressos por meio do valor médio do NAS* e número absoluto de avaliações entre parênteses. Porto Alegre, RS, Brasil, 2023

**Mês**	**UTI** ^‡^ **A**	**UTI** ^‡^ **B**	**UTI** ^‡^ **C**	**UTI** ^‡^ **D**	**UTI** ^‡^ **E**	**Total**
Janeiro	87,1 (233)	81,2 (242)	82,7 (229)	85,7 (243)	87,1 (121)	84,5 (1.068)
Fevereiro	81,2 (188)	84,8 (246)	82,4 (210)	86,2 (208)	84,3 (106)	83,8 (958)
Março	83,0 (207)	86,6 (270)	82,9 (245)	87,9 (234)	84,8 (122)	85,2 (1.078)
Abril	85,6 (215)	83,1 (236)	86,1 (218)	89,3 (239)	90,0 (132)	86,6 (1.040)
Maio	86,9 (203)	84,7 (299)	88,0 (250)	90,6 (242)	86,8 (132)	87,4 (1.126)
Junho	85,1 (180)	83,1 (234)	86,4 (240)	96,4 (212)	87,2 (128)	87,6 (994)
Julho	83,7 (218)	83,4 (280)	87,3 (246)	94,0 (228)	88,9 (120)	87,2 (1.92)
Agosto	83,7 (200)	86,6 (305)	80,8 (259)	91,9 (261)	88,9 (135)	86,3 (1.160)
Setembro	84,8 (227)	81,7 (235)	80,6 (258)	89,3 (236)	88,5 (108)	84,4 (1.094)
Total	84,6 (1.871)	84,0 (2.377)	84,1 (2.155)	90,1 (2.103)	87,5 (1.104)	85,9 (9.610)

*NAS = *Nursing Activities Score*; ^†^UTIs = Unidades de Terapia Intensiva; ^‡^UTI = Unidade de Terapia Intensiva

**Tabela 2:** - Valores de NAS* e demais elementos para os Cálculos I e II do dimensionamento de profissionais de enfermagem entre janeiro e setembro de 2023. Cálculo II (a) refere-se a cinco turnos de trabalho, enquanto Cálculo II (b) trata de quatro turnos. Porto Alegre, RS, Brasil, 2023

**Mês**	NAS*				**Cálculo I**			**Cálculo II (a) - 5 turnos**			**Cálculo II (b) - 4 turnos**		
	**Total pontuação mês**	**Total pontuação dia**	**Total pontuação dia x 14,4**	NAS* médio (%)	**THE** ^†^	**QP** ^‡^ **= THE** ^†^ **x KM ^§^ (Equipe total)**	**Quadro de enfermeiros**	**Pontuação no dia/100 x 5 equipes**	**Mais 15% (Equipe total)**	**Quadro de enfermeiros**	**Pontuação do dia/100 x 4 equipes**	**Mais 15%** (Equipe total)**	**Quadro de enfermeiros**
Janeiro	90.250	3.008	43.320,00	84,5	722,00	161	84	150	173	90	120	138	72
Fevereiro	80.289	2.676	38.538,72	83,8	642,31	144	75	134	154	80	107	123	64
Março	91.795	3.060	44.061,60	85,2	734,36	164	85	153	176	91	122	141	73
Abril	90.023	3.001	43.211,04	86,6	720,18	161	84	150	173	90	120	138	72
Maio	98.367	3.279	47.216,16	87,4	786,94	176	91	164	189	98	131	151	78
Junho	87.098	2.903	41.807,04	87,6	696,78	156	81	145	167	87	116	134	69
Julho	95.181	3.173	45.686,88	87,2	761,45	170	89	159	182	95	127	146	76
Agosto	100.068	3.336	48.032,64	86,3	800,85	179	93	167	192	100	133	153	80
Setembro	92.329	3.078	44.317,92	84,4	738,63	165	86	154	177	92	123	142	74
Média dos meses	91.711	3.057	44.021,33	85,9	733,68	164	85	153	176	91	122	140	73

*NAS = *Nursing Activities Score*; ^†^THE = Total de Horas de Enfermagem; ^‡^QP = Quadro de Pessoal; ^§^KM = 0,2236 (Constante de Marinho, coeficiente deduzido sobre as horas e os dias semanais trabalhados e o Índice de Segurança Técnica)

## Discussão

No presente estudo, utilizando-se o NAS como o principal componente de cálculos, foi possível dimensionar o pessoal recomendado para um CTI de 45 leitos, a partir de duas equações, sendo uma delas, ainda, desmembrada em duas possibilidades, de acordo com o número de turnos de trabalho. Enquanto o Cálculo I demonstrou serem necessários, em média, 164 profissionais, o Cálculo II, quando se considera a presença de cinco ou de quatro turnos, determinou 176 ou 140 trabalhadores, respectivamente.

Verificou-se que, a despeito do cálculo empregado, ao longo dos meses, a variação de estimativa do quadro de pessoal foi de 30 ou mais profissionais. Especialmente, ao se empregar o Cálculo II para cinco turnos, a diferença entre os meses de maio e agosto foi de 38 profissionais. Dentre as 9.610 avaliações do NAS realizadas nas cinco UTI do estudo, o NAS médio foi de 85,9%, correspondendo a 20,2 horas de cuidados de enfermagem/paciente nas 24 horas do dia, um número superior a outros estudos brasileiros^(26-27)^ e também de países como Itália^(28)^, Portugal^(10)^ e Nova Zelândia^(29)^. Essa estimativa de carga de trabalho diária difere também da normativa nacional vigente sobre dimensionamento de enfermagem, que parametriza 18 horas de cuidado/dia aos pacientes considerados de cuidado intensivo^(19)^. Tais divergências podem ser explicadas devido à heterogeneidade das UTIs e às características dos pacientes internados.

Já foi relatado que pacientes críticos internados em hospitais públicos apresentam média do NAS de admissão maior (68,1%) comparados aos internados em hospitais privados (56%)^(30)^. O CTI do presente estudo atende, predominantemente, pacientes complexos provenientes do Sistema Único de Saúde. Ademais, o lugar é centro de referência regional para diversas especialidades, incluindo transplantes, o que pode explicar o maior valor de NAS em relação a outros hospitais públicos brasileiros.

De fato, o valor total do NAS encontrado neste estudo apresentou variação ao longo dos meses, visto que o maior escore médio foi observado no mês de maio (98.367), enquanto isso, a menor pontuação foi encontrada nos meses de fevereiro e agosto (80.289). Similarmente, uma série histórica italiana que acompanhou 5.856 pacientes ao longo de cinco anos incluiu 28.390 avaliações e identificou variação do NAS ao longo dos semestres, de modo que o mínimo foi: 61,0±17,1%; e o máximo: 68,6±17,4%^(28)^.

Sendo assim, determinar, por meio de estudos longitudinais robustos, se a variação documentada no presente estudo se deve ao acaso ou se ela se repetirá ao longo dos anos pode contribuir fortemente para o planejamento mais racional da distribuição da força de trabalho, por exemplo, na provisão de férias e de períodos com maior reforço quantitativo de trabalhadores.

A variabilidade na estimativa do quadro de pessoal de enfermagem obtida ao se aplicar cada um dos métodos, assim como as duas variações do Cálculo II para cinco ou quatro turnos de trabalho, não se explicam pela variação do NAS, uma vez que o escore do NAS não diferiu na operacionalização das equações. Isso significa que a equação/método de cálculo para dimensionar a equipe de enfermagem em UTI tem impacto direto na definição numérica e categórica da composição do quadro de profissionais. Ou seja, não se trata de uma questão de “meramente” definir o instrumento de aferição da carga de trabalho, visto que em ambos os casos isso foi concentrado no NAS, mas também de observar qual será o processamento de dados empregado. Esta é a contribuição direta deste estudo.

A principal diferença entre os dois cálculos testados de forma pioneira neste estudo se deve ao fato de que a primeira procedeu à transformação da média do NAS da unidade em horas de enfermagem, conforme parâmetros do instrumento original (cada ponto equivalente a 14,4 minutos)^(21-22)^ e, com isso feito, baseou-se na equação já preconizada pelo COFEN^(17)^ para unidades hospitalares à época do estudo. Por sua vez, o Cálculo II baseou-se na recomendação de se prever um profissional a cada 100 pontos do NAS, o que também está de acordo aos pressupostos originais do instrumento^(21-22)^. Além disso, os resultados do cálculo dependiam do número de equipes de enfermagem atuantes na UTI dimensionada.

Apesar de ser legítimo e necessário reconhecer a inovação do método de Cálculo II, acredita-se que o Cálculo I é o método mais adequado por três motivos: 1) ele se fundamenta de forma mais direta nos pressupostos normativos da entidade de classe no país; 2) é intermediário, considerando os valores mínimos e máximos encontrados na pesquisa, portanto, indicando razoabilidade; 3) não incorre na possibilidade de estabelecer o quantitativo total de trabalhadores claramente discrepante ao se considerar o número de turnos de trabalho na equação, desfavorecendo equipes de enfermagem que se dividem em quatro turnos, o que é mais comum no cenário brasileiro.

Mesmo considerando que o Cálculo I seja mais adequado e que, a partir dele, tenham sido utilizados elementos normalizados pelo COFEN, a metodologia proposta neste estudo não é diretamente referida por este órgão, tanto em 2017^(17)^ como em 2024^(19)^, o que justifica a necessidade de maior aprofundamento metodológico sobre planejamento de pessoal de enfermagem em terapia intensiva, e que isso avance em termos legais. Outro ponto a ser assumido é que usando a metodologia I, as horas previstas em regime contratual serão consideradas^(17)^, o que (in)direta, mas não tão incisivamente, tende a repercutir no número de equipes.

Alocar profissionais de enfermagem em turnos é uma necessidade para a organização do trabalho hospitalar ininterrupto. Sobre isso, o Cálculo II tende a privilegiar equipes organizadas em cinco turnos quando considerado o número total de trabalhadores dimensionados, assim como o quantitativo de enfermeiros. Entretanto, se divididos entre turnos e retirando-se 15% das coberturas de ausências que não são distribuídas em escala de trabalho^(31)^, o número total de profissionais de enfermagem por turno (n=30) seria equivalente se os trabalhadores fossem divididos tanto em quatro quanto em cinco equipes.

A informação acima é relevante quando se pensa em alocação da força de trabalho de enfermagem atrelada a elementos orçamentários. Evidentemente, o número de pessoal para compor quatro equipes é menor do que cinco, e, consequentemente, se hipotetizado um incentivo remuneratório equivalente, setores atuantes com quatro equipes seriam economicamente mais interessantes à sustentabilidade das organizações. Apesar da possível vantagem sob a ótica financeira, adiciona-se que trabalhadores de enfermagem de UTI distribuídos em cinco equipes comparados a quatro possuem melhores condições laborais, maior satisfação com o trabalho e menor percepção de carga de trabalho^(32)^. Tais resultados tendem a trazer desfechos clínicos favoráveis aos pacientes e ainda podem repercutir em economia para as organizações de saúde^(1)^.

A pesquisa^(23)^ realizada em uma UTI do Paraná aplicou o NAS em 107 pacientes, obtendo uma média 697,3 pontos na unidade de oito leitos. Para determinar a proporção de enfermeiros sobre a equipe, as autoras empregaram o parâmetro proposto pelo COFEN da época do estudo. Com isso, elas verificaram que o quadro de profissionais de enfermagem disponível estava defasado e que a proporção de enfermeiros não atendia à recomendação do Conselho (35,7% *vs* 52,5%)^(23)^. Importa ressaltar que o estudo citado é o que desenvolveu o Cálculo II, empregado na presente pesquisa, e que a proporção de enfermeiros no quadro de pessoal dimensionado da época era discretamente superior ao que se recomenda na normativa vigente na época do presente estudo^(17)^, mantendo-se após “atualização” em 2024^(19)^. Essa redução pode refletir a resistência dos serviços de saúde em contratar profissionais de nível superior, o que é preocupante, uma vez que cabe à entidade de classe representar os interesses da profissão, além de garantir coerência com os pressupostos deontológicos da enfermagem.

Há mais de três décadas, o COFEN define que os enfermeiros são os profissionais melhor habilitados para prestar cuidados de alta complexidade a pacientes em risco de vida^(12)^, como aqueles internados em UTI. Por outro lado, a mesma entidade estabelece que 52% dos profissionais que prestam cuidados em UTI devem ser enfermeiros^(19)^. Nesse sentido, ainda parece ser necessário equacionar o evidente distanciamento entre essas duas diretrizes, o que reforça a importância de maior aprofundamento metodológico sobre o planejamento de pessoal de enfermagem em UTI, configurando uma contribuição deste estudo.

No âmbito da terapia intensiva, a regulamentação técnica que estabelece critérios de funcionamento desses serviços, entre outros, aborda a distribuição da força de trabalho de enfermagem^(11)^. Todavia, ela diverge do que se preconiza na lei do exercício profissional^(12)^ e na normativa da categoria sobre dimensionamento de pessoal de enfermagem^(19)^. Tal contradição demanda ações de fortalecimento do aparato legal e da fiscalização, no que se refere ao quantitativo mínimo de trabalhadores de enfermagem.

Embora seja validado para uso no Brasil^(21)^ e internacionalmente reconhecido^(7,9-10)^, o NAS foi derivado de um cenário diferente do nacional^(22)^. Aspectos relacionados à organização do trabalho da enfermagem brasileira precisam ser considerados, uma vez que o NAS refere-se a “profissional de enfermagem” – algo usual para países em que não há divisão técnica e hierárquica da categoria. Revisão sistemática recente que incluiu 23 estudos observacionais realizados nos Estados Unidos da América, Austrália, Bélgica, China, Coreia do Sul e Reino Unido concluiu que, apesar do viés metodológico presente em alguns dos artigos incluídos no estudo, a contratação de enfermeiros ao invés de profissionais técnicos, além de adequação no quantitativo de pessoal, melhora os desfechos clínicos e os custos hospitalares^(33)^.

O NAS, assim como outras ferramentas desenvolvidas para aferição da carga de trabalho de enfermagem, não considera variáveis como a estrutura física dos serviços e a dinâmica de trabalho específica de cada área assistencial (neste caso, cada UTI), além da experiência e do nível de competência profissional. Apesar disso, o estudo de coorte que comparou a carga de trabalho da enfermagem aferida pelo NAS em relação ao diagnóstico de COVID-19 confirmou que o grupo de pacientes positivo para o diagnóstico apresentou maior carga de trabalho^(8)^, o que pode ser uma evidência de que, embora revisões sejam profícuas, o NAS continua sensível para captar oscilações na demanda/dependência de assistência e horas de enfermagem em UTI. Portanto, atende aos ideais da concepção do instrumento^(21-22)^.

Mesmo que uma escala estime que o grau de dependência de cuidados de enfermagem de dois pacientes possibilite que ambos sejam cuidados por um único profissional de enfermagem, na prática, isso será viabilizado exclusivamente se ambos encontrarem-se em leitos lado a lado, ou com uma proximidade que possibilite que o profissional de enfermagem visualize e monitorize simultaneamente a ambos os pacientes. Em outras palavras, a distribuição da força de trabalho de enfermagem, além do aparato instrumental, também precisa da experiência e do bom-senso dos enfermeiros e gestores.

O presente estudo se propôs a trazer à luz algumas discussões sobre o dimensionamento e a composição do pessoal de enfermagem para o cuidado ao paciente crítico em UTI, apreciando procedimentos metodológicos de viabilizar esses fins. Apesar do grande número de observações, limita-se a alguns meses do ano e em um único centro, o que pode influenciar os valores de NAS e das estimativas de quantitativo de pessoal. Por outro lado, a pesquisa documenta a necessidade de estudos que contribuam para a compreensão sobre os componentes do cálculo de pessoal de enfermagem, a fim de estabelecer uma relação de pacientes por profissional segura e, ao mesmo tempo, viável. Enfermeiros devem ser protagonistas nessas e em outras definições gerenciais sobre o exercício profissional. Entidades representativas da classe têm a missão de dar suporte e amparo para isso.

## Conclusão

O presente estudo demonstrou o dimensionamento de pessoal de enfermagem recomendado para um CTI de 45 leitos, a partir de dois cálculos, sendo um deles desmembrado em duas possibilidades conforme o número de turnos de trabalho. O NAS foi utilizado como um dos elementos centrais nos cálculos apresentados. Sua estabilidade reforçou a possibilidade de utilizá-lo para este fim.

Conclui-se que os métodos de cálculo demonstrados geram resultados nitidamente discrepantes, especialmente considerando o número absoluto total de profissionais (quer seja de nível médio ou superior) deduzido pelas equações. Ao comparar os dois cálculos, acredita-se que o Cálculo I seja mais adequado, uma vez que não é afetado tão diretamente pela distribuição de turnos de trabalho, que não é uma constante na organização do trabalho da enfermagem. Portanto, a atual normativa brasileira vigente sobre o dimensionamento de pessoal de enfermagem em terapia intensiva carece de maior aprofundamento metodológico sobre a previsão de pessoal de enfermagem no contexto de UTI.
